# An Emergent Network for the Diffusion of Innovations Among Local Health Departments at the Onset of the COVID-19 Pandemic

**DOI:** 10.5888/pcd18.200536

**Published:** 2021-03-04

**Authors:** Minwoong Chung, Debra Dekker, Chelsea Gridley-Smith, James W. Dearing

**Affiliations:** 1 Department of Communicology, University of Hawaii at Manoa, Honolulu, Hawaii; 2 National Association of County and City Health Officials, Washington, District of Columbia; 3 Department of Communication, Michigan State University, East Lansing, Michigan

## Abstract

**Introduction:**

Communication networks among professionals can be pathways for accelerating the diffusion of innovations if some local health departments (LHDs) drive the spread of knowledge. Such a network could prove valuable during public health emergencies such as the novel coronavirus disease 2019 (COVID-19) pandemic. Our objective was to determine whether LHDs in the United States were tied together in an informal network to share information and advice about innovative community health practices, programs, and policies.

**Methods:**

In January and February 2020, we conducted an online survey of 2,303 senior LHD leaders to ask several questions about their sources of advice. We asked respondents to rank up to 3 other LHDs whose practices informed their work on new public health programs, evidence-based practices, and policies intended to improve community health. We used a social network analysis program to assess answers.

**Results:**

A total of 329 LHDs responded. An emergent network appeared to operate nationally among 740 LHDs. Eleven LHDs were repeatedly nominated by peers as sources of advice or examples (ie, opinion leaders), and 24 acted as relational bridges to hold these emergent networks together (ie, boundary spanners). Although 2 LHDs played both roles, most LHDs we surveyed performed neither of these roles.

**Conclusion:**

Opinion leading and boundary spanning health departments can be accessed to increase the likelihood of affecting the rate of interest in and adoption of innovations. Decision makers involved in disseminating new public health practices, programs, or policies may find our results useful both for emergencies and for practice-as-usual.

**Summary**

**What is already known on this topic?**

Leaders of local health departments (LHDs) look to other LHDs for sharing information and opinions about innovative community health practices, programs, and policies.

**What is added by this report?**

Our data show that during the early stages of the current novel coronavirus disease 2019 (COVID-19) pandemic, small proportions of LHDs were repeatedly nominated by peers as sources of advice or example, whereas others acted as relational bridges to hold the network together. Most LHDs in our data performed neither of these roles.

**What are the implications for public health practice?**

Advice-seeking methods such as those reported here, if used in dissemination strategy, may increase the consideration and adoption of best practices that otherwise can take years to diffuse into public health practice.

## Introduction

Organization leaders regularly look to similar leaders and organizations for cues and insights about innovations and for appropriate responses to external opportunities and threats. Sometimes leaders are reacting to coercive processes such as government policy enactment; other times they react voluntarily on the basis of social influence or because of perceived norms (1). One of the foundations of diffusion is that innovations flow most easily among people who perceive similarity with each other and among organizations of similar type. Collective rejection of innovations results from the same principle (2). This “birds of a feather” effect is a reason why professional and trade associations can be powerful pathways of diffusion among association members (3). They link together people and organizations with commonalities. In combination, these linkages function as relational networks that can be identified, accessed, and engaged as an essential lever in public health preparedness (4). Knowledge of such directional ties can be useful for reimagining a strengthened public health system of the future (5).

How communication networks among leaders of local health departments (LHDs) are structured has rarely been reported. Such networks are social systems (6), the members of which share a relational structure. Some members are more important to the system’s functioning than others; some engage in systems to various degrees (7). An ideal characteristic of public health practitioners is an appreciation for such social systems and an understanding that communication is the currency that flows through them (8).

Network analysis is especially well-suited to the study of communication networks and has been used to assess the structural and communicative dimensions of relationships among public health professionals and public health organizations (9) and among their leaders (10). Leaders of public health organizations who are members of professional and trade associations are an important stratum that can be accessed and engaged for advancing public health (11). The study of advice seeking, in particular, can illuminate who looks to whom for good ideas to improve health (12).

The main objective of our study was to learn whether certain LHDs play outsized roles as opinion-leading health departments and whether some departments function to tie the country together and reach remote LHDs in one large, informal public health network. Interorganizational networks that do this — a small proportion of opinion-leading organizations and organizations that span boundaries within the network — are actionable for intervention because they offer an existing and efficient pathway for accelerating the diffusion of effective innovations.

## Methods

 We developed a brief sociometric (who-to-whom) survey and pretested it with leaders of the National Association of County and City Health Officials (NACCHO). NACCHO routinely collects data from LHD leaders on topics such as the state of local emergency preparedness (13). The institutional review board of Michigan State University determined that this study was exempt from regulations, because there were no foreseeable reasonable risks to study participants.

NACCHO sent an email announcement of a forthcoming survey to a senior leader in each of 2,303 US LHDs in mid January 2020. One week later on January 22, an email message was sent with an embedded link to a consent form and the survey. Three follow-up reminders were sent once a week. The survey closed on February 10. Recipients were instructed that a person with decision-making responsibility for adopting new public health programs, evidence-based practices, and policies intended to improve community health and with the title of chief health officer, director of population health, deputy health officer, or medical director should complete the survey. Respondents identified their own LHD and then listed up to 3 other LHDs “whose example you look to or follow with respect to new public health programs, evidence-based practices, and policies intended to improve community health.” They were asked to list the LHDs in the order of their importance to them, as the most–, second-most–, and third-most–valued source of advice. We collected basic demographic information on LHD leaders (ie, gender, years worked in their present LHD, years worked as a health professional) at the end of the survey.

Surveys were returned by 546 respondents. If a respondent did not nominate at least one advice source, that survey was considered unusable. After discarding unusable surveys, 329 surveys were included for analysis (329/2,303; 14%); 230 surveys (73%) were completed by women and 86 surveys (27%) were completed by men. The mean (SD) number of years worked in their present LHD was 13.5 (10.2), and the mean (SD) number of years worked as a public health professional was 25.1 (11.2). We found no significant difference in these demographics between leaders who nominated and those who did not nominate their advice sources (*P* > .05). Of the 217 discarded surveys, 50 participants provided their demographics: 39 of them were women (78%) and 11 of them were men (22%). The mean (SD) number of years they had worked in their present LHD was 14.8 (13.5), and the mean (SD) number of years they had worked as a health professional was 24.6 (11.1).

The 329 respondents identified 493 unique advice sources. Because some advice sources were not respondents, we derived data about more LHDs than just the number of respondents, as is typical with questionnaire-based social network analysis. In our case this yielded 740 unique LHDs (social network “nodes”), tied together through 863 reported advice-seeking relationships (social network “ties”).

We used urban–rural commuting area codes to assess the representation of urban and rural LHDs in our sample (14). Of the 329 usable surveys, 180 (55%) were from leaders of urban LHDs, and 149 (45%) were from leaders of rural LHDs. These percentages appear to mirror those of the population of LHDs (N = 2,303) whose leaders were sent the invitation: 1,262 were urban LHDs (55%) and 1,041 were rural LHDs (45%). Of the 217 discarded surveys, 113 (52%) were from urban LHD leaders and 104 (48%) were from rural LHD leaders. We saw no significant difference between urban and rural LHD leaders with respect to their survey completion, χ^2^(1, N = 546) = 0.37, *P* = .55.

Network data were analyzed at national, US Census division (eg, New England, Middle Atlantic), and LHD levels by using SPSS (IBM Corp) and UCINET (Analytic Technologies). LHD opinion leadership was measured as nodal in-degree centrality scores, that is, the number of nominations of a department by survey respondents. For example, if a health department was named by 6 survey respondents as an advice source, it would have an in-degree score of 6.

LHD boundary spanning was measured as nodal betweenness centrality scores. Betweenness assesses the degree to which an LHD lies on the shortest relational path connecting every 2 nodes in the network (ie, the shortest route between any 2 nodes in a social network map). Boundary spanners in social networks are nodes (organizations, in our case) that function to tie the network together; they connect disparate nodes that are members of different groups within an overall network. For example, a department that lies on the shortest paths between 12 department-to-department dyads would have a betweenness score of 12.

We determined which LHDs were opinion leading or boundary spanning on the basis of departments that fell 2 SDs above the mean on each metric (ie, in-degree centrality scores for opinion leaders and betweenness centrality scores for boundary spanners) (12). Network visualizations were created with Gephi (15).

## Results

We calculated network analysis results for the 740 local health departments in the data set by 1) the number of advice seekers, advice sources, and ties nationally and for each US Census division, and 2) the in-degree scores (mean [SD]) of opinion-leading and other LHDs, betweenness centrality scores (mean [SD]) of boundary-spanning and other LHDs at both national and US Census division levels (Table 1). Nationally, 84% of all reported advice-seeking ties were within census division relationships. Our analysis showed that of these740 LHDs in the United States, 11 qualified as opinion-leading organizations, and 24 LHDs qualified as boundary-spanning organizations. The 11 opinion-leading LHDs had a mean of 12.2 nominations from other LHDs nationally. Nonopinion-leading LHDs had an in-degree mean score of 1.0. The highest number of nominations for a single LHD was 38. Two hundred and forty-seven LHDs received no nominations (in-degree score = 0). Boundary-spanning LHDs — those departments with high betweenness centrality scores — were also substantially different from nonboundary-spanning LHDs, with national mean scores of 17.2 for boundary-spanning and 0.3 for nonboundary-spanning.

**Table 1:** Characteristics of Advice-Seeking Local Health Departments in the United States, by US Census Division, January–February 2020^a^

Characteristic	National	New England	Middle Atlantic	East North Central	West North Central	South Atlantic	East South Central	West South Central	Mountain	Pacific
**Nodes, no.^b^**	740	99	55	205	194	72	18	63	58	50
Advice seekers	329	40	20	88	80	24	7	26	22	22
Advice sources	493	64	38	142	138	51	13	46	40	32
**Ties^c^**	863	101	53	239	211	64	19	64	54	58
Interdivisional ties, no.	140	14	9	39	24	17	5	9	19	4
Interdivisional ties, %	16.2	13.9	17.0	16.3	11.4	26.6	26.3	14.1	35.2	6.9
**Opinion-leading local health departments^d^**										
No.	11	5	4	6	10	4	1	3	3	2
In-degree, nonopinion leaders, mean (SD)	1.0 (1.1)	0.8 (.7)	0.8 (.7)	1.0 (.9)	0.9 (.7)	0.8 (.6)	0.9 (.7)	0.9 (.6)	0.8 (.6)	0.9 (0.9)
In-degree, opinion leaders, mean (SD)	12.2 (9.3)	6.0 (2.9)	3.0 (0)	7.0 (2.9)	5.0 (1.3)	3.0 (0)	1.1 (1.0)	4.3 (1.2)	3.3 (0.6)	6.5 (2.1)
**Boundary-spanning local health departments^e^**										
No.	24	3	3	5	11	3	1	6	4	3
Betweenness centrality, nonboundary spanners, mean (SD)	0.3 (1.2)	0 (0.3)	0 (0)	0.3 (1.3)	0.3 (1.0)	0 (0)	0.2 (1.0)	0.1 (0.3)	0 (0)	0 (0.2)
Betweenness centrality, boundary spanners, mean (SD)	17.2 (9.0)	10.3 (3.1)	5.7 (0.6)	21.1 (8.1)	12.7 (5.7)	2.7 (0.6)	12.0 (0)	4.1 (1.3)	3.5 (1.0)	3.5 (1.3)

a Derived from 329 respondents to a survey of 2,303 local health departments conducted from January 22 through February 10, 2020 through the National Association of County and City Health Officials.

b Nodes refer to local health departments in the United States. Advice seekers refer to the departments who look to other departments for advice or examples for their work on new public health programs, evidence-based practices, and policies intended to improve community health. Advice sources refer to the departments whose practices inform others’ work.

c Ties refer to advice-seeking relationships. Interdivisional ties are advice-seeking relationships between local health departments in different US Census divisions.

d Opinion leaders are the influential local health departments whose practices inform the work of many other local health departments. They were identified by the number of nominations (ie, in-degrees) by survey respondents as an advice source. The local health departments whose number of nominations fell 2 SDs above the mean were identified as opinion leaders.

e Boundary spanners are the local health departments that link disparate departments in different groups within an overall network. They were identified based on their betweenness centrality scores (ie, the degree to which a department lies on the shortest relational path connecting every 2 local health departments in the network) in our data set. The local health departments whose betweenness centrality scores fell 2 SDs above the mean were identified as boundary spanners.

**Figure 1:**
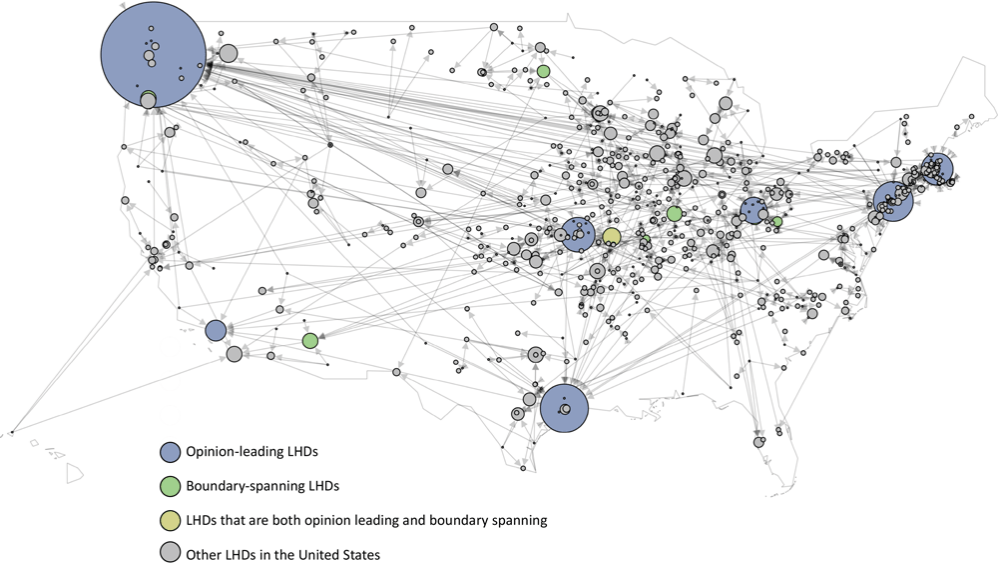
Advice-seeking networks among local health departments (LHDs) in the United States identified in a survey of 329 health departments through the National Association of County and City Health Officials, 2020. The size of a node reflects the opinion leadership (number of nominations received) of each LHD. Lines indicate advice-seeking relationships between an LHD and others, and arrowheads show the direction of advice-seeking. Number of lines indicate number of LHDs seeking advice from an LHD.

When visualized, both opinion-leading LHDs and boundary-spanning LHDs were prominent features of a national informal advice-seeking network among the 740 LHDs for which we had data (Figure 1). Although several LHDs played outsized roles as opinion leaders for many others, many nodes with just a few ties were sources of advice or examples for others, including some LHDs in rural areas of the country.

**Figure 2:**
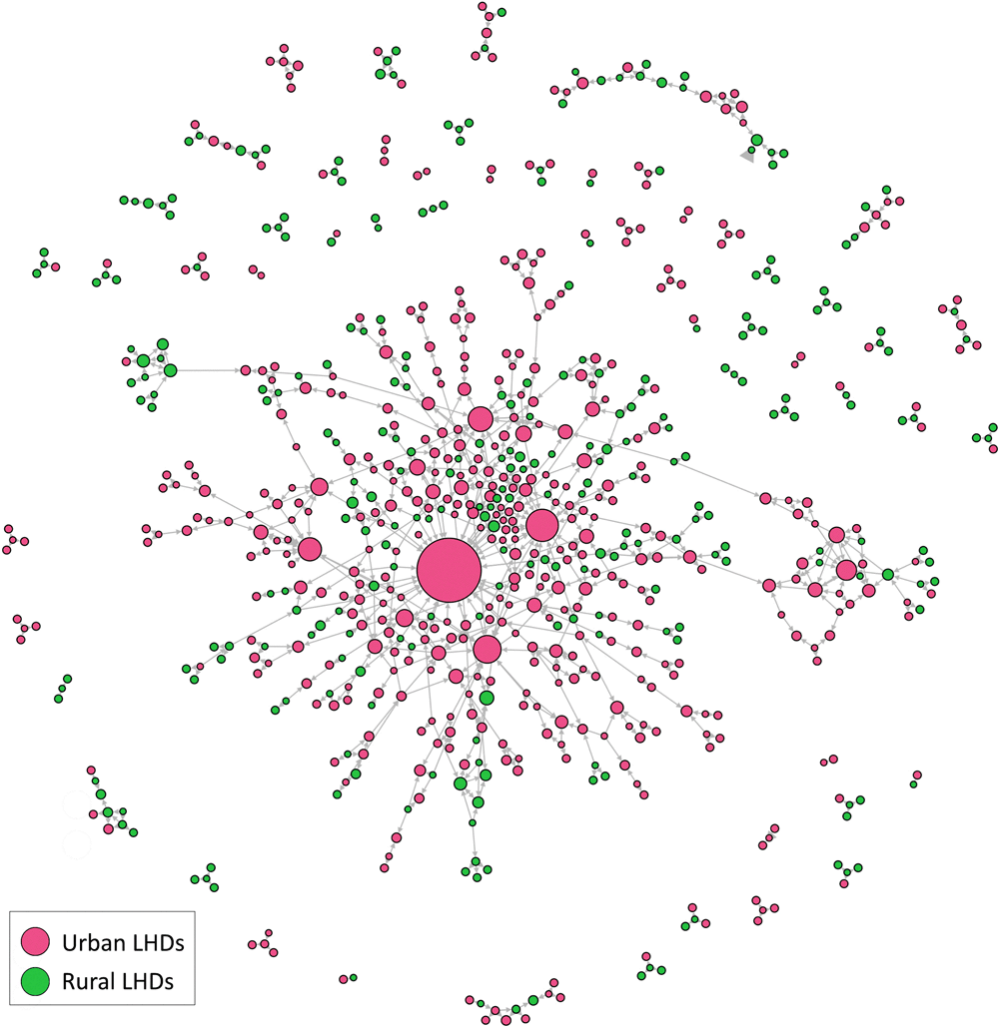
Urban and rural representation of local health departments (LHDs). Node size reflects the number of nominations an LHD received from senior leaders of other LHDs. The distinction was based on the rural–urban commuting area codes retrieved from the US Department of Agriculture (14). Node size corresponds to number of in-degree ties.

To understand the extent to which opinion leadership was concentrated in large urban departments or co-existed in rural areas, we analyzed the rural–urban classification of opinion-leading LHDs and the extent to which opinion seekers and opinion sources were present in rural or urban areas. Of the 740 LHDs, 456 (62%) were in urban areas and 284 (38%) were in rural areas. Of the 863 ties between these LHDs, 471 (55%) emanated from urban LHDs and 392 ties (45%) emanated from rural LHDs. Urban departments (n = 426) nominated urban departments 90% of the time, and rural LHDs (n = 163) nominated rural departments 42% of the time. We found 1 large, interconnected group of LHDs around urban departments and many other very small, disconnected groups of rural and urban departments, which suggests the advice-giving prominence of urban LHDs for many other urban and rural departments (Figure 2).

We ranked the 10 highest-scoring opinion-leading and boundary-spanning LHDs with their respective in-degree and betweenness centrality scores (Table 2). Although 2 LHDs played both roles, most LHDs we surveyed performed neither of these roles.

**Table 2:** Ten Highest Ranked Opinion-Leading and Boundary-Spanning Local Health Departments, United States, January–February 2020^a^

Local Health Department	Score^b^
**Opinion leaders**	
Public Health – Seattle and King County (Washington State)	38
Harris County Public Health (Texas)	17
New York City Department of Health and Mental Hygiene (New York)	14
Boston Public Health Commission (Massachusetts)	12
Kansas City Health Department (Missouri)	12
Delaware General Health District (Ohio)	9
Los Angeles County Department of Public Health (California)	7
Cambridge Public Health Department (Massachusetts)	7
Union County Health Department (Ohio)	6
Columbia/Boone County Health Department (Missouri)	6
**Boundary spanners**	
Maricopa County Department of Public Health (Arizona)	41
Union County Health Department (Ohio)	33
Champaign-Urbana Public Health District (Illinois)	30
Clark County Public Health (Washington)	26
Franklin County Health District (Ohio)	25
Fargo/Cass Public Health (North Dakota)	24
Madison County Health Department (Illinois)	24
Columbia/Boone County Health Department (Missouri)	21
Noble County Health Department (Ohio)	21

a Derived from 329 respondents to a survey of 2,303 local health departments conducted from January 22 through February 10, 2020 through the National Association of County and City Health Officials. Opinion leaders are influential local health departments whose practices inform the work of many other local health departments on new public health programs, evidence-based practices, and policies intended to improve community health. Boundary spanners are local health departments that link disparate departments in different groups within an overall network.

b For opinion leaders, in-degree scores were used; in-degree scores refer to the number of nominations of a local health department by survey respondents as an advice source. For boundary spanners, betweenness centrality scores were used; betweenness centrality scores refer to the degree to which a department lies on the shortest relational path connecting every 2 LHDs in the network.

## Discussion

We asked whether leaders of the nation’s local health departments look to each other for informing their work about new public health programs, evidence-based practices, and policies intended to improve community health. We also investigated whether such relationships extend across the United States. Results suggest that a small proportion of LHDs serve as models for many others, and that certain departments tie together a national LHD advice-seeking network. Results such as these offer insights for researchers, policy makers, and practitioners who wish to spread new and worthy practices, programs, and policies. Change agent effectiveness is associated with how much time that agent spends with influential social system members when introducing and discussing how to use innovations (16). Social network data represent an important lever in diffusion of innovations (17).

Our study has limitations, and these data require caution in interpretation. The usable response rate was 14% (329/2,303). Respondents were allowed to nominate no more than 3 LHDs. Data were sought from only 1 respondent per department. Although these limits were intended to reduce respondent burden, the actual informal advice-seeking network that ties together LHDs in the United States is likely much larger, denser, and more topically multifaceted than is represented here. Our results can be considered a very conservative estimate of network structure and the relationships that comprise it. For example, senior leaders are not the only people in their departments who know the reputations of other LHDs and may seek advice from them. The January 22, 2020, release of our survey invitation occurred 2 days after the first confirmed case of COVID-19 in Snohomish County, Washington (18), still weeks before the severity of the pandemic in the United States was apparent. Possibly the early attention of local health officials to COVID-19 both depressed the survey response rate and led to the prominence of the Seattle and King County Health Department, which in nonpandemic times may also be looked to by officials in other LHDs. Yet during the early days of the pandemic, probably both longstanding ties and new inquiries to health departments in jurisdictions struck first by a crisis might be reflected in data collection such as ours. Thus, although the wording of our questions did not refer to emergency situations, respondents probably answered about both credible peer departments and those with direct experience with a new, serious threat — Seattle and King County in this instance.

Our study of advice seeking as a means for understanding how to efficiently intervene in a professional network is a type of formative evaluation that can be joined with other ways of gaining insight into who affects whom for drawing attention to new practices, programs, and policies. Other methods for gaining insight include professional judgment, firsthand experience, and research methodologies such as correlational studies and observation of interactions among public health officials and other health department staff members. Data such as ours can be leveraged to augment decision making about how to disseminate the results of research and accelerate the adoption of public health interventions, during routine times or those characterized by responses to a pandemic. Our results suggest how to disseminate new public health information, practices, programs, and policies efficiently, especially when LHD directors may consider those innovations to be consequential and controversial. For instance, given that opinion leaders are considered experts on a given issue and trustworthy, the dissemination of innovations from opinion-leading LHDs can propel the rate of consideration and adoption when compared with dissemination from other organizations. Boundary-spanning LHDs can help to move innovations from group to group across a network, which is often a greater challenge for change agents than achieving adoption by the nodes within a group.
